# Recent Trends in Enzyme‐Based Biosensors for On‐Site Screening of Antibiotic Residues

**DOI:** 10.1002/asia.202401409

**Published:** 2025-07-31

**Authors:** Sapna Sudan, Bin Xie, Sunil Bhand

**Affiliations:** ^1^ Biosensor Lab, Department of Chemistry BITS Pilani KK Birla Goa Campus Vasco da gama Goa 403726 India; ^2^ Pure and Applied Biochemistry Lund University Lund S 22100 Sweden

**Keywords:** Antibiotic residues, Antimicrobial resistance, Biosensors, Enzymes

## Abstract

Antibiotics are used to treat both humans and animals for both preventive and therapeutic purposes. The overreliance on and misuse of antibiotics has given rise to a stealth pandemic, known as antimicrobial resistance (AMR). Globally, they pose a significant threat to human, animal, and environmental health. Surveillance of antibiotic residues in the environment, especially in wastewater, for tackling AMR can be a potent way to tackle deaths associated with AMR. Aquatic ecosystems are ideal habitats for the dissemination of AMR because they are frequently impacted by anthropogenic activities. Nearly half of the world's population resides in rural areas, which lack the infrastructure and resources necessary to manage wastewater effectively and sustainably. In the past decade, there has been no significant addition of new antibiotics. In this review, we discuss emerging enzyme‐based biosensor technologies for on‐site and rapid determination of antibiotics in the environment, with a focus on optical, electrochemical, and thermometric methods of detection.

## Graphical abstract

Antibiotic pollution pathways and biosensor intervention for effective monitoring and control of antimicrobial resistance.

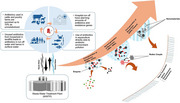

## 1 Introduction

**Figure 1:**
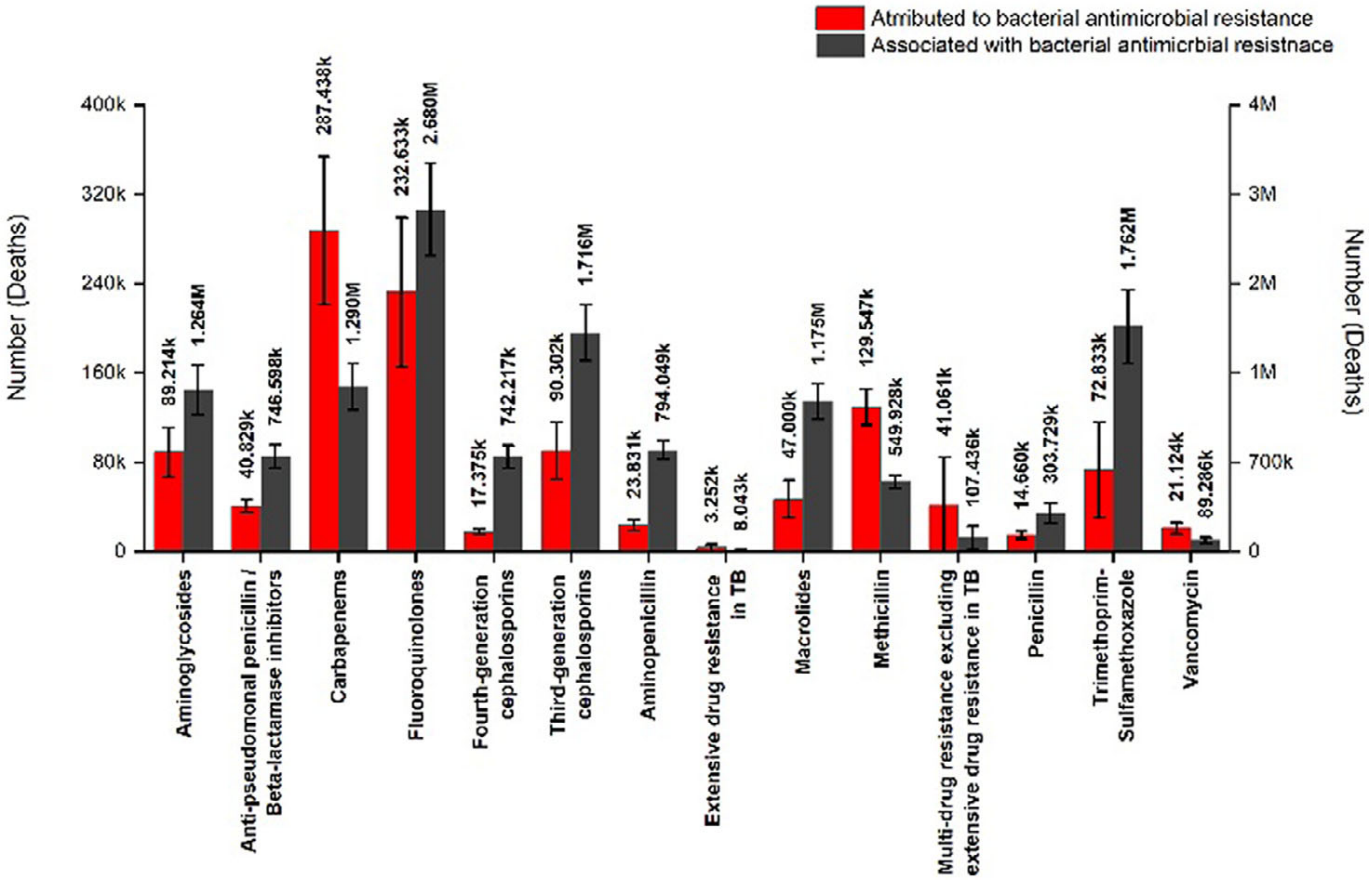
Deaths in 2021, showed that approximately one million fatalities were attributed to AMR, while 1.27 million deaths were directly associated with AMR. Data obtained from Global Health Data (GHDx), Institute for Health Metrics and Evaluation 2021.^[^ ^2^, ^3^ ^]^

**Figure 2:**
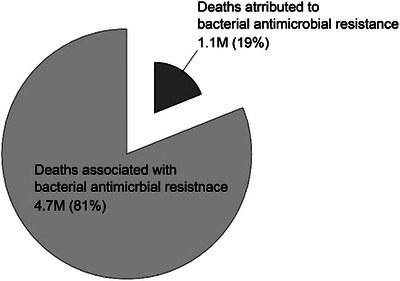
Number of deaths due to pathogens attributed to antibiotic resistance. Data obtained from Global Health Data (GHDx), Institute for Health Metrics and Evaluation 2021.^[^ ^2^, ^3^ ^]^

AMR is defined as the loss of susceptibility to a particular class of antibiotics. When a resistant bacterial strain causes infection, it tends to be untreated and potentially lethal. Without effective antibiotics, interventions such as surgery, cancer therapy, organ transplantation, and infections acquired in the community can become lethal, causing millions of deaths annually. Emergence of AMR is a natural phenomenon; however, the misuse and overuse of antibiotics has accelerated this phenomenon and it has become one of the most critical threats to public health and the environment. About a million fatalities were attributed to AMR, while 1.27 million deaths were directly associated with AMR (Figures 1 and 2). AMR disproportionately affects rural communities, in particular of Low and Middle Income countries where 70‐80% population is rural, due to social, economic and health system vulnerabilities.^[^ ^1^, ^2^, ^3^ ^]^

It is predicted that AMR can lead to 10 million deaths annually^[^ ^2^, ^3^, ^4^ ^]^ and a report by world bank mentions 28.3 million people will be pushed into poverty.^[^ ^5^, ^6^ ^]^ Early qualitative determination of AMR can help provide better treatment regimens to the patient, keep track of resistance for precision in later diagnosis, reduce the burden on medical facilities, and provide personalised antibiotic treatment, further preventing the emergence of AMR. It has been reported that subtherapeutic does of antibiotics can be found in surface water bodies,^[^ ^7^, ^8^, ^9^, ^10^ ^]^ reported to exceed the maximum residual limit (MRL) in water bodies in low‐ and middle‐income countries.^[^ ^11^ ^]^

**Figure 3:**
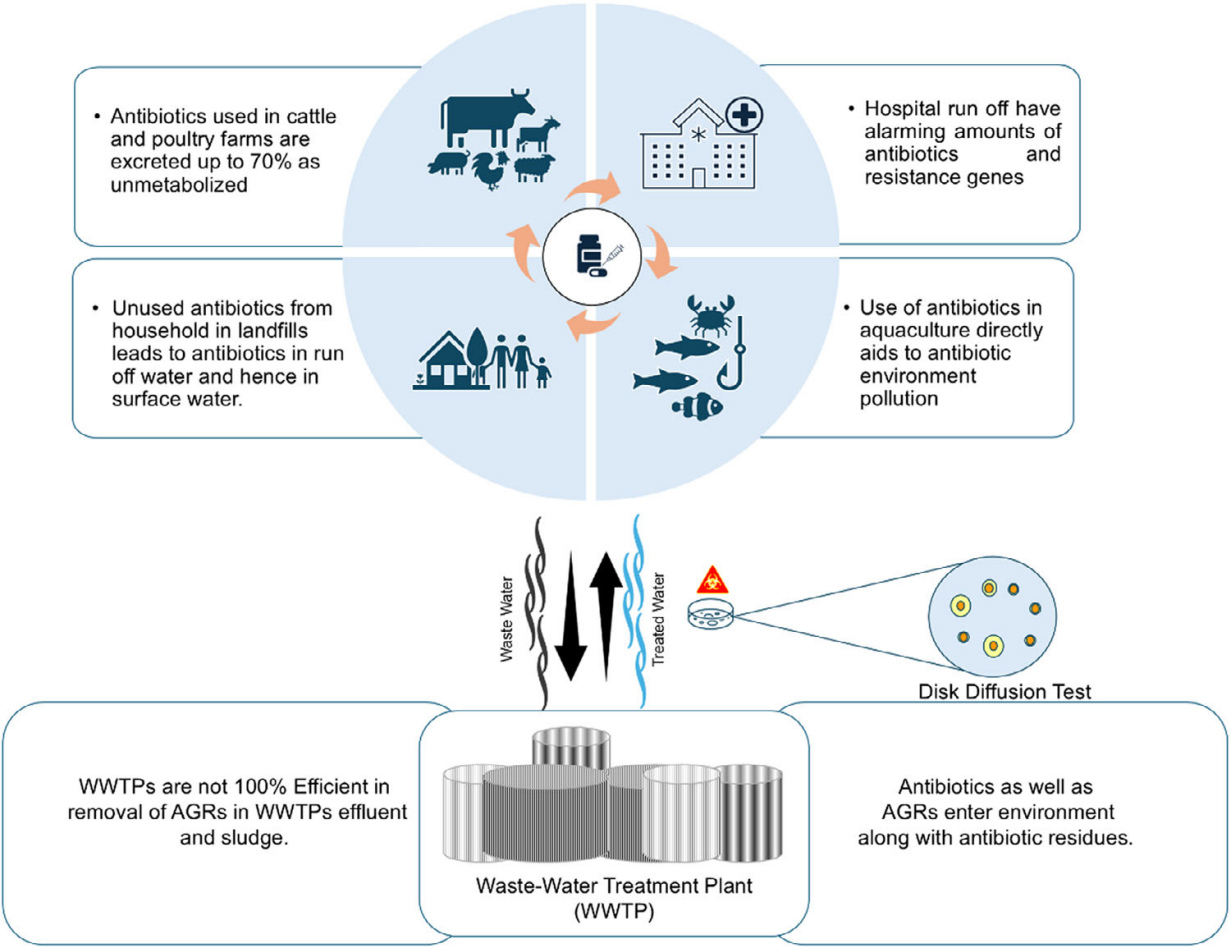
Various sources of antibiotics in water bodies.

There are various ways antibiotics end up in water bodies (Figure 3). Antibiotics are frequently used in aquaculture,^[^ ^12^, ^13^, ^14^, ^15^ ^]^ agriculture,^[^ ^16^, ^17^, ^18^ ^]^ dairy and poultry health management^[^ ^19^, ^20^, ^21^, ^22^, ^23^, ^24^, ^25^, ^26^, ^27^, ^28^, ^29^, ^30^, ^31^ ^]^ and in human healthcare.^[^ ^32^, ^33^ ^]^ Most of the times antibiotics enter the environment in unchecked disposal of antibiotics from households, hospitals, particularly in low‐ and middle‐ income countries results disposal of the waste water into rivers owing to the inefficiency of wastewater treatment (WWT) plants.^[^ ^34^, ^35^, ^36^, ^37^, ^38^, ^39^, ^40^, ^41^ ^]^

Water plays a significant role in transforming and moving contaminants, such as drug residues, antibiotic‐resistant bacteria, and genes, especially in places subjected to heavy anthropogenic activity.^[^ ^42^, ^43^, ^44^, ^45^ ^]^ Additionally, climate change and environmental pollution are exacerbating AMR.^[^ ^46^ ^]^ Antibiotic‐contamination pushes bacteria either pathogenic or non‐pathogenic, under selection pressure. Alongside Co‐pollutants and microplastics in water facilitate the spread of AMR.^[^ ^47^ ^]^

The development of antibiotic resistance by pathogenic bacteria has been a bottleneck in antibiotic therapy since its introduction; however, this process has accelerated manyfold over the past few decades owing to the misuse and overuse of antibiotics of antibiotics have accelerated AMR many‐fold and poses a risk to effective tools to trace trends and defences against the danger of AMR.

The detection of antibiotic residues is crucial for tracking and predicting resistance in a particular community/region to ensure treatment with suitable antibiotics and to prevent the acceleration of AMR. Quantification of antibiotic residues in water bodies is generally performed using mass spectrometry (LC/MS) in conjunction with liquid chromatography. However, this involves high cost, complex operations, trained personnel, and sample preparation. Therefore, there is a dire need for rapid, inexpensive, user‐friendly, and point‐of‐care devices for the qualitative and quantitative determination of antibiotics.

**Figure 4:**
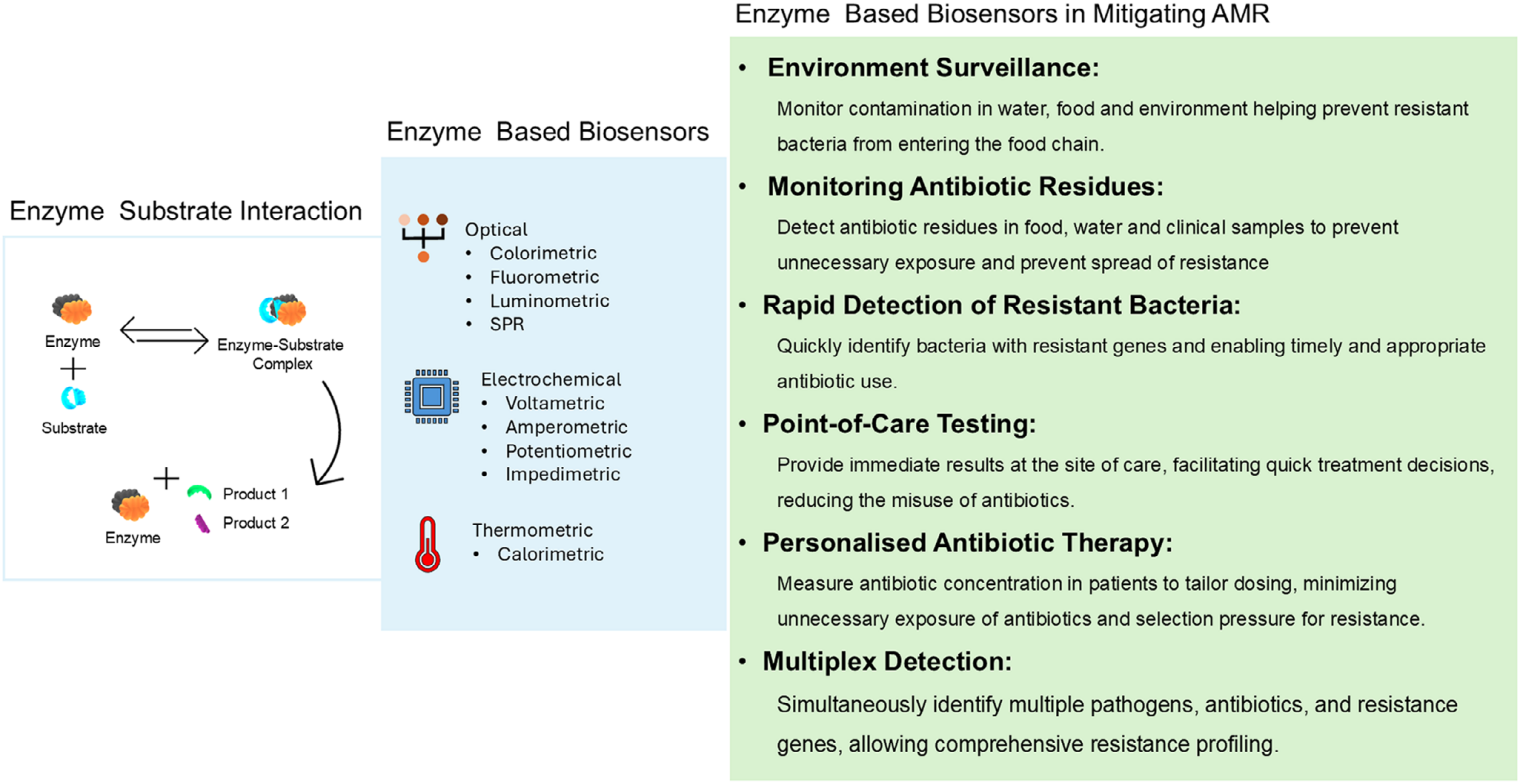
Application of enzyme‐based Biosensors in mitigating AMR.

Conventionally, antibiotics are determined by traditional thin‐layered chromatography (TLC), high‐performance liquid chromatography (HPLC), liquid chromatography‐mass spectrometry (LC‐MS), and microbial culture‐based techniques. These techniques are labour intensive, require trained personnel to operate the machines, and are time‐consuming, requiring at least 12–24 h. Hence, affordable and precise screening techniques with low detection thresholds and high sample throughputs are ideal. Biosensors present a significant advancement over conventional analytical methods across multiple fields, including environmental monitoring due to their high sensitivity and selectivity,^[^ ^48^ ^]^ rapid and real‐time detection,^[^ ^49^ ^]^ portability, minimal sample requirements, reusability^[^ ^50^ ^]^ and multiplexing capabilities,^[^ ^51^ ^]^ ease of use and potential for cost effective mass production. These advantages make biosensors especially valuable for point‐of‐care diagnostics, real‐time monitoring, and suitable for application where immediate, reliable results are essential (Figure 4).

## 2 Enzyme‐Based Biosensors

IUAPC defines a biosensor as a device that can detect chemical moieties mainly through optical, electrical, or thermal signals through a particular biochemical process mediated by immunosystems, tissues, organelles, or entire cells termed biorecognition elements. Usually, in a biosensor, the biorecognition element is immobilised on the transducer surface, which specifically interacts with the target analyte without additional chemical treatment and produces physiochemical changes on the transducer surfaces.^[^ ^52^, ^53^ ^]^

These changes were measured using a transducer to quantify analyte concentration. Considering the type of signal transduction method, biosensors can be broadly classified into optical biosensors, electrochemical biosensors, and calorimetric biosensors. They can also be classified based on the interaction of the analyte with the biorecognition element: *catalytic biosensors (enzyme‐based)*, in which the interaction leads to the formation of a new product, and *affinity biosensors*, in which the interaction is specific to the analyte.

**Figure 5:**
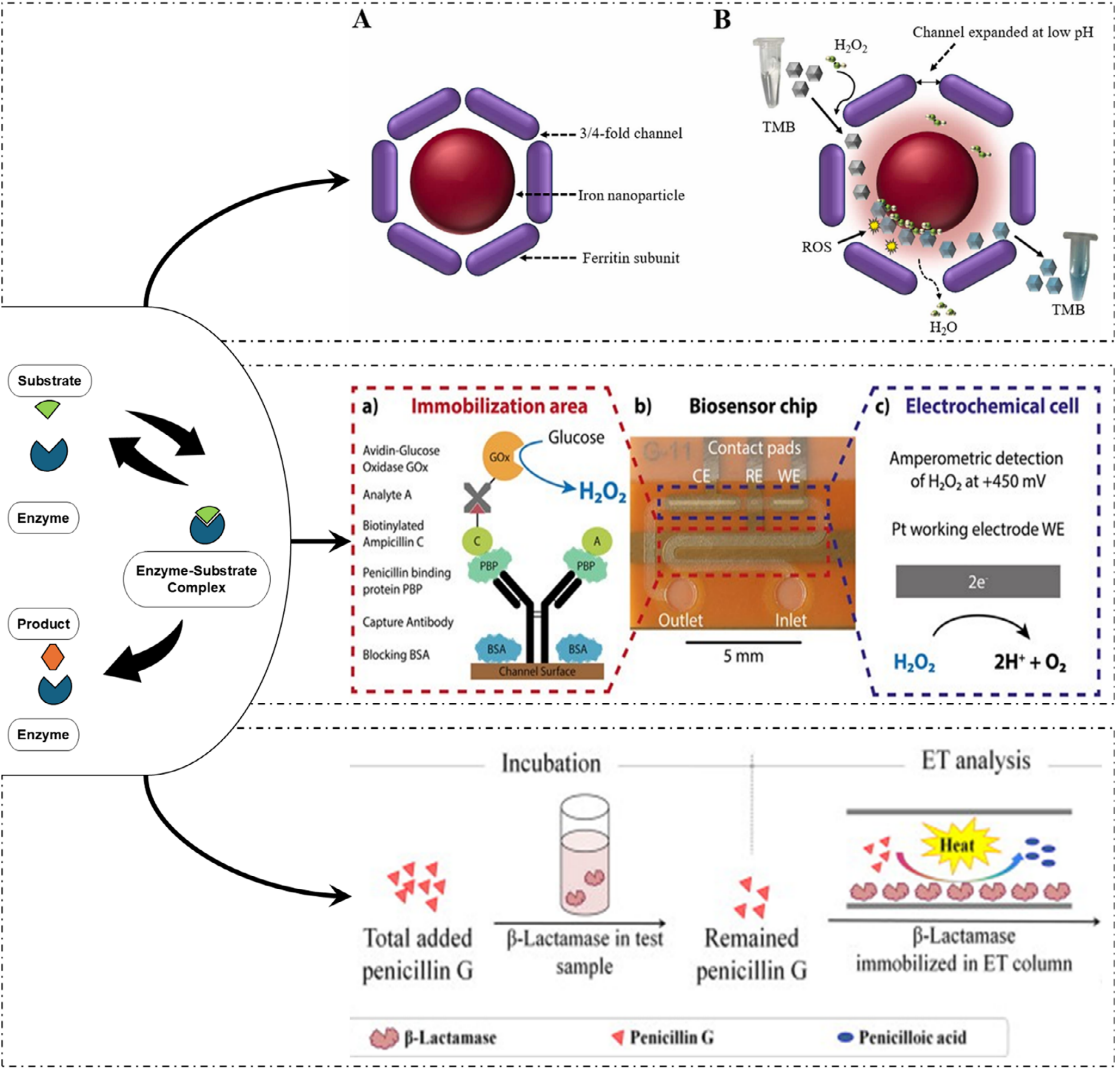
Illustration represents various types of enzyme‐based biosensors; (1) Schematic of an enzyme reaction; (2) A,Illustrates the structure of Bovine Spleen Ferritin (BSF), and B; Peroxidase mimicking activity of BSF for optical detection of tetracycline Adapted with permissiom.^[^ ^54^ ^]^ Copyright Elsevier, 2023; (3) Illustration of electrochemical microfluidic platform: (a) Schematic of competitive β‐lactam assay based on PBP3 receptor and GOx enzyme. (b) Photograph of microfluidic integrated biosensing platform consisting of Counter electrode CE, the reference electrode RE and the working electrode WE. (c) Illustrates oxidation of hydrogen peroxide produced at Pt WE for amperometric detection inside the electrochemical cell. Adapted with permission.^[^ ^55^ ^]^ Copyright 2017,Nature; (4) Schematics of (a) hydrolysis of β‐lactam ring in Penicillin G by β‐lactamase and (b) principle of thermometric analysis procedure. Adapted with permission.^[^ ^56^ ^]^ Copyright 2013, Elsevier.

The sensing principle of enzymatic biosensors usually involves the detection of either the consumption of a substrate (analyte) or formation of a product over time as the pH changes, the release of gases such as H_2_, CO_2_, NH_3,_ etc., or changes in the intensity or wavelength of light and heat emission, which occur as a result of the breakdown of the analyte molecule or assembly of analyte molecules in the presence of an enzyme (Figure 5). The transducer surface converts these changes into readable signals to identify and quantify the analyte concentrations. In this review, we discuss enzymatic biosensors for the preliminary screening of antibiotic residues. Current advances and challenges in developing point‐of‐care enzymatic biosensors.

Enzymes are biological catalysts that accelerate biochemical reactions. The catalytic efficiency of the enzymes was studied and measured using a laboratory method called enzyme assay. Enzyme assays are used to study enzyme kinetics and inhibition and play crucial roles in analytical assays with clinical significance. As catalysts, enzymes are only required at low concentrations and are not consumed in the reaction.^[^ ^57^ ^]^ Enzyme specificity toward its substrate and high biocatalyst turnover rates makes it a highly competitive element when choosing biorecognition element for a biosensor. Enzyme reactions can be studied primarily using optical, electrochemical, or thermo‐electrochemical transduction methods.

There are two ways to utilise enzymes as biorecognition elements, direct and indirect. In the direct method, the detector has direct access to physical or chemical changes during the enzymatic reaction and does not require any label for detection.^[^ ^58^ ^]^ The sensing technique which requires a secondary element and the bio recognition element (label) to bind to the analyte. However, the stability of enzymes outside their natural environment and the recovery of enzymes after the completion of the reaction are hurdles in the development of enzymatic biosensors. Immobilisation of the enzyme onto/inside a support matrix on the transducer surface provides a solution for enzyme recovery after biosensor applications. Immobilisation not only imparts reusability to the biosensor but, in many cases, enhances its activity over a more significant temperature and pH range.^[^ ^59^, ^60^, ^61^ ^]^

### 2.1 Optical Biosensors

Optical biosensors are a popular choice for detection of antibiotic residues because they enable the real‐time detection of various biomarkers with high sensitivity and selectivity, cost‐effectiveness, and portability. Optical biosensors utilize bioreceptors like enzymes, antibodies, aptamers that are immobilized on sensor surface to enable selective binding of the target antibiotics. These binding events are measured as optical signals such as color, fluorescence or refractive index, enabling real‐time detection of antibiotics even at low concentrations. In context of AMR optical biosensors can determine the minimum inhibitory concentration (MIC) of antibiotics against susceptible/resistant pathogens providing early identification of resistant infections, which is critical for timely intervention and effective treatment. They enable both qualitative and quantitative analysis of antibiotics, supporting application in clinical diagnostics, environmental monitoring and food safety.

Advanced optical biosensors with multiplexing and high‐throughput capabilities can be engineered to measure changes in optical properties in response to the metabolic activity of pathogen in the presence of antibiotics, allowing rapid determination of MIC and resistance profile within clinically relevant timeframes. Further integration with Internet of Things (IoT) platforms makes it possible to have personalised monitoring of AMR diagnosis and antibiotic administration.

Currently, various enzyme‐based optical biosensors are available for antibiotic detection. These well‐established enzyme‐based optical biosensing strategies for antibiotic detection can be divided into the following biosensing strategies as summarized in Table 1.

- Colorimetric Biosensor
- Fluorometric Biosensor
- Luminometric Biosensor
- Surface Plasmon Resonance Biosensor

**Table 1:** Some of the reported enzyme‐based optical biosensors.

Biosensor type	Enzyme	Analyte	LOD ^a^ ^)^	Matrix	Label	Response Time	Ref.^b^ ^)^
Colorimetric	β‐galactosidase	Paromycin Tetracycline Chloramhenicol Erythromycin	0.5 µg/mL 2.1 µg/mL 0.8 µg/mL 6.1 µg/mL	Spiked environmental water samples for Paromycin only	Chlorophenol red β‐galactopyranoside	–	^[^ ^63^ ^]^
	Bovine Spleen Ferritin	Tetracycline Oxytetracycline Chlortetetracyline Doxycycline	15.0 nM 21.6 nM 20.2 nM 20.1 nM	Pork meat	3,3′,5,5′‐Tetramethylbenzidine	–	^[^ ^54^ ^]^
	TetX2	Tetracycline	60 nM	Milk	Thionine	6 min	^[^ ^62^ ^]^
Fluorimetric	β‐lactamase	β‐ lactams	10 nM	–	BADAN	60–70 sec	^[^ ^64^ ^]^
	Pen P‐E166Cf/N170Q	β‐ lactams	1 µM	Mouse serum, Milk	Fluorescein	–	^[^ ^65^ ^]^
	Nicking Enzyme (Nt. Alwl)	Kanamycin	1.23 pM	Milk	Fluorescent probe	40 min	^[^ ^66^ ^]^
Luminescent	β‐lactamase	Ampicillin Amoxicillin Ceftazidime Cefotaxime	0.18 nM (milk) 9 nM (meat)	Wastewater Milk Chicken meat	–	–	^[^ ^67^ ^]^
Surface Plasmon Resonance	Carboxy peptidase	Penicillin G	1.2 ug/Kg	Milk	Peptide	–	^[^ ^68^ ^]^

^a)^ Limits of detection

^b)^ Reference

#### 2.1.1 Colorimetric Biosensors

**Figure 6:**
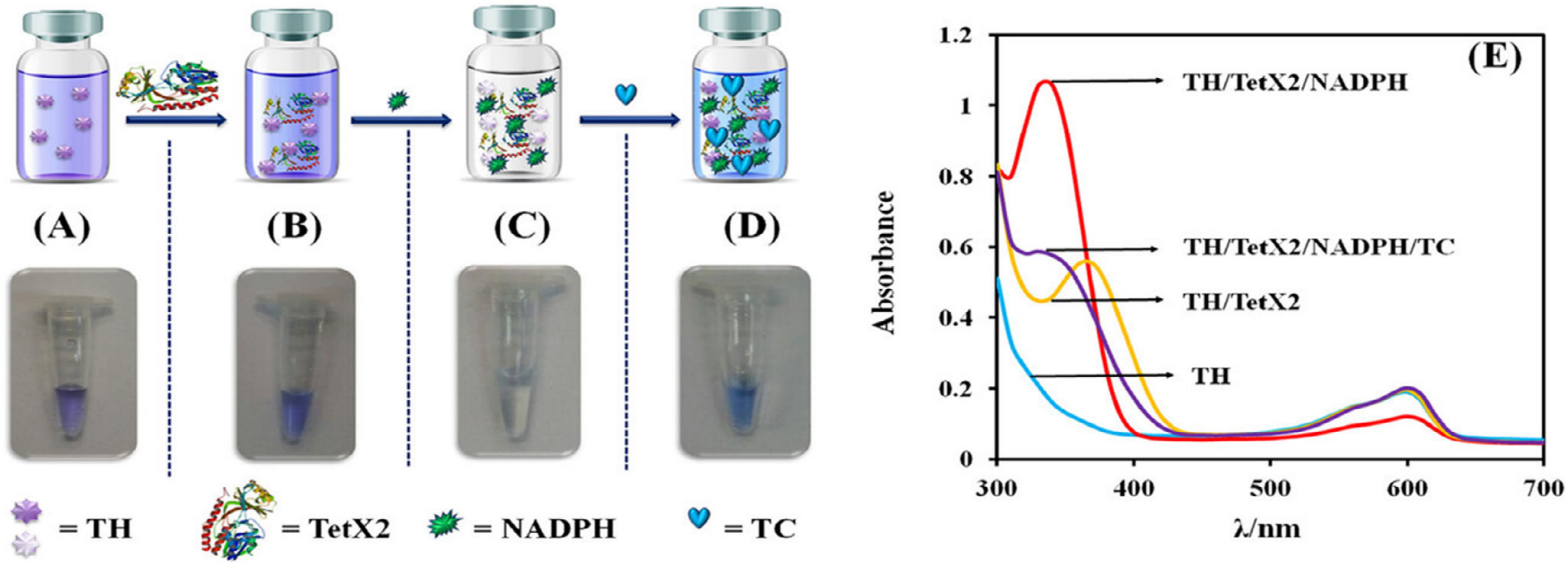
Sensing mechanism for the TetX2‐induced TH‐based colorimetric assay in Na_2_HPO_4_ solution (0.1 M, pH 8.5): (A) TH‐ox solution (1 mg/mL), intitiaaly appears purple/blue color. (B) When 3 µL of TetX2 (Conc. 4 mg/mL) is added to TH‐ox solution, there is no color change. (C) Adding NAD(P)H (1 mM) to TH/TetX2 solution the color shift from purple/blue to colorless.within 30 s of pippetting. (D) The detection solution after incubation with 7 µM TC, which generated a blue color in < 2 Min. (E) UV–vis spectra of TH‐ox, TH/TetX2, TH/TetX2/NAD(P)H, and TH/TetX2/NAD(P)H/TC immediately after imaging to illustrate the changes. Adapted with permission.^[^ ^62^ ^]^ Copyright 2022, Wiley.

**Figure 7:**
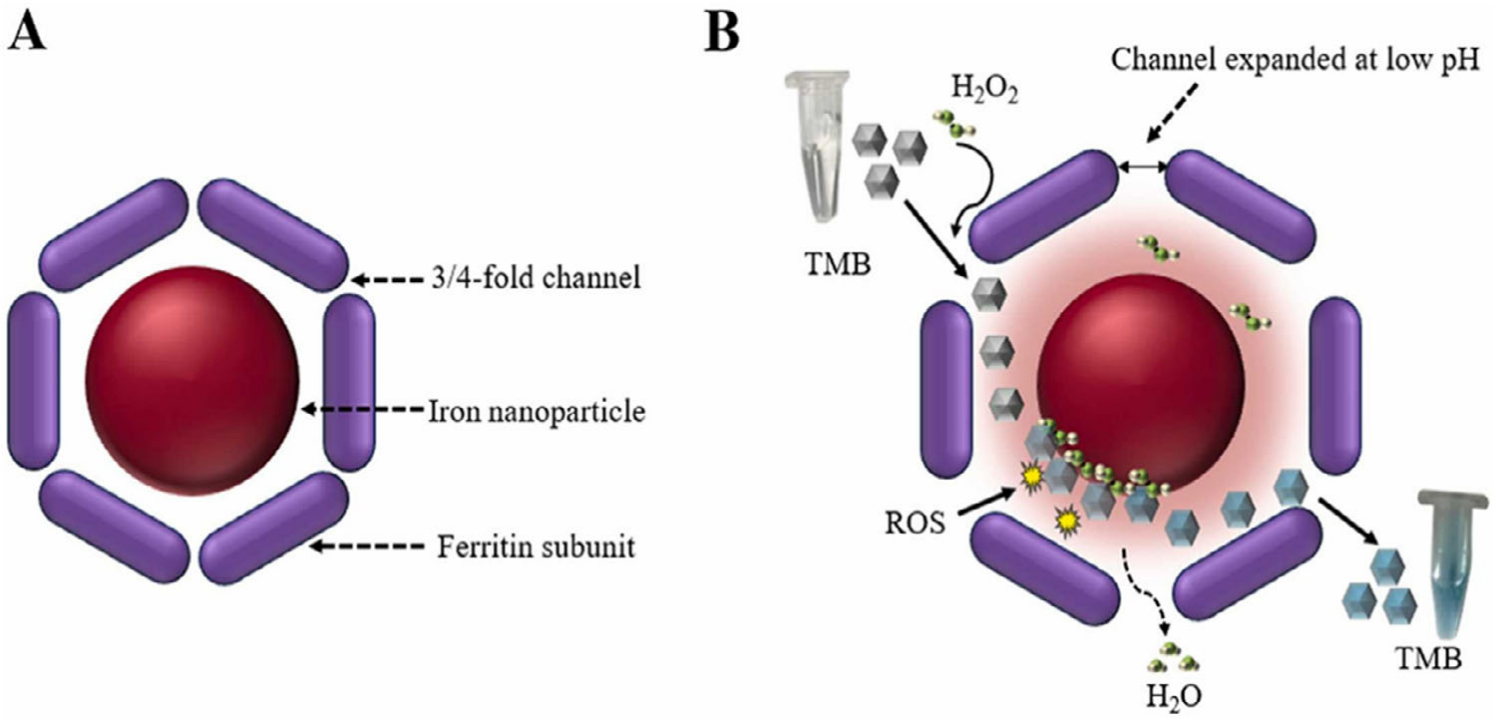
(A) Illustrates the structure of Bovine Spleen Ferritin (BSF) and (B) peroxidase mimicking activity of BSF. Adapted with permission.^[^ ^54^ ^]^ Copyright 2023, Elsevier.

Many researchers are developing colorimetric protocols for easy detection of antibiotics in environmental matrices. Researchers have developed a simple, paper based colorimetric biosensor for detection of antibiotics that inhibit the bacterial protein synthesis. Freeze dried paper discs containing In Vitro Transcription/Translation (IVTT) system, LacZ DNA template and Chlorophenol red β‐galactopyranoside (CPRG) substrate were rehydrated with antibiotic solutions and incubated at 37 °C for 2 h. β‐galactosidase (β‐gal) synthesis from DNA template is inhibited in presence of antibiotics, reported a paper‐disc enzyme β‐gal based biosensor for detection of antibiotics.^[^ ^63^ ^]^ The biosensor provides semi‐quantitative detection with naked eyes and quantitative detection when combined with a desktop scanner and image analysis software. As reported, a biosensor for highly sensitive detection of tetracycline antibiotics utilised the enhances catalytic activity of Bovine Spleen Ferritin with 3,3′,5,5′‐tetramethylbezidine (TMB) as mediator (Figure 7).^[^ ^54^ ^]^.Another bacterial enzyme TetX2 in presence of NAD(P)H and thionine(TH) as indicator for activity of a to detect Tetracycline as low as 60 nM and within 6 min of time in milk (Figure 6).^[^ ^62^ ^]^ The biosensor have selectivity for tetracycline and visual detection by the naked eyes. The colour change was monitored by images using phone camera and image analysing software

#### 2.1.2 Fluorometric Biosensors

Fluorescence is a more sensitive technique than colorimetry. Fluorescent assays can utilise either the inherent fluorescence of an analyte molecule or labelled fluorescent molecule. The study reports development of highly sensitive thermostable fluorescent biosensor for detecting β‐lactam antibiotics in food and clinical samples. The biosensor is based on a mutant of *Bacillus licheniforms* PenP β‐lactamase engineered for enhanced stability and sensitivity with Fluorescein dye covalently attached to the enzyme's active site, allowing a “turn‐on” fluorescent biosensor when it bind to β‐lactam antibiotics.^[^ ^65^ ^]^ Researchers further engineered two biosensors E166Cb and E166Cb/N170Q by covalently binding active site of PenP β‐lactamase mutant with 6‐Bromoacetyl‐2‐dimethylaminonaphthalene (BADAN).^[^ ^64^ ^]^ The modified active site of β‐lactamase enzyme is developed as a turn‐on fluorescent biosensor for various β‐lactam antibiotics. As a result of enzyme engineering E166Cb/N170Q showed better results due to reduced catalytic activity of antibiotics. Another study demonstrated a aptasensor that uses an enzyme powered 3D DNA walker for detection of kanamycin. High density DNA substrate coupled with a fluorescent probe, the walking track, is activated in presence of kanamycin. A nicking enzyme (Nt. Alwl) cleaves the DNA amplifying the signal to detect as low as 1.23 pM of kanamycin.^[^ ^66^ ^]^

#### 2.1.3 Luminescent Biosensor

The authors developed a rapid, point‐of‐care optical biosensor based on enzymatic hydrolysis and nanostructured polyaniline‐coated optical fibres.^[^ ^67^ ^]^ The sensor detects β‐lactam antibiotics by measuring the changes in absorbance at 435 nm caused by enzymatic breakdown of antibiotics, which alters the chemical state of polyaniline nanofibers.

#### 2.1.4 Surface Plasmon Resonance (SPR) Biosensors

A study reports the development of two surface plasmon resonance (SPR)‐based biosensor assays for detecting β‐lactam antibiotics in milk. The assays utilise the enzymatic activity of carboxypeptidase, which converts a 3‐ peptide to a 2‐peptide‐ a process inhibited by the presence of β‐lactam antibiotics.^[^ ^68^ ^]^ Detection is achieved by measuring either the enzymatic product or the remaining substrate using specific antibodies.

### 2.2 Electrochemical Biosensors

In the past decade, electrochemical devices have become popular for the development of biosensors because of their enhanced sensitivity making them suitable for monitoring trace residues in clinical, environmental and food samples. Incorporation of nanomaterials like graphene, carbon nanotubes and other metallic nanoparticles enhance the surface area, conductivity and hence sensitivity of the biosensors. Furthermore, advanced electrode fabrication techniques allow for miniaturizing, multiplexing and integration with hand held devices like smart phones. A list of enzyme based electrochemical biosensors with relevant figures of merit is presented in Table 2.

Electrochemical enzyme‐based sensors are pioneers in the development of commercially available biosensors^[^ ^69^ ^]^ and can be divided into the following types.

- Voltametric biosensor
- Amperometric biosensors
- Impedimetric biosensors

#### 2.2.1 Voltametric Biosensor

Voltametric biosensing relies on measuring the current flow through the working electrode immersed in electroactive species, which is analysed by varying the potential.^[^ ^71^ ^]^ The Gold (Au) L‐Cystine‐Platinum (PT)‐Penicillinase nanowire array enables sensitive, stable, and simultaneous detection of penicillin and tetracyline in mixtures, with performance tuneable by adjusting segment length.^[^ ^72^ ^]^ A second generation electrochemical was developed for detection of penicillin G in milk, utilising a carbon paste electrode modified with class b β‐lactamase and cobalt phthalocyanine as an electron mediator.^[^ ^73^ ^]^ A reuseable electrochemical biosensor was developed by electropolymerizing methylene blue (MB) on a glassy carbon electrode New Delhi Metal‐β‐lactamase‐1 (NDM‐1)^[^ ^74^ ^]^ to detect penicillin and ampicillin in milk (Figure 10). A highly sensitive Penicillin G biosensor was successfully fabricated by immobilizing Pen‐X enzyme on a gold biochip, and tested in a [K_3_ Fe(CN)_6_]∖PBS system.^[^ ^75^ ^]^ The biosensor demonstrates high sensitivity, a low detection limit of 1.26 nM, good stability and reproducibility at room temperature. Using mesoporous carbon sphere @UiO‐66‐NH2 (MSC@UiO‐66‐NH2) core shell composite embedded laccase enzyme.^[^ ^76^ ^]^ The MSC@UiO‐66‐NH2 composite allows effective enzyme immobilization, prevents inactivation, and enhances the stability and activity compared to free enzyme system. The developed biosensor was able to detect tetracycline in spiked honey and milk samples. A carbon paste electrode (CPE) modified with multiwalled carbon nanotubes (MWCNTs) and horse radish peroxidase (HRP) utilizing the electrocatalytic oxidation of oxytetracycline mediated by H_2_O_2_.^[^ ^77^ ^]^ Enzyme stability is essential for biosensor development in commercial applications. It is impacted by temperature, and pH of the surrounding, concentration of the enzyme and metal ion inhibitors.^[^ ^78^, ^79^ ^]^ A voltametric biosensing platform for detection of Penicillin G in dairy samples is fabricated by immobilizing Class B β‐lactamase enzyme on a carbon paste electrode with cobaltpthalocyanine as electron mediator for improved response time of 60 s.^[^ ^73^ ^]^

**Figure 8:**
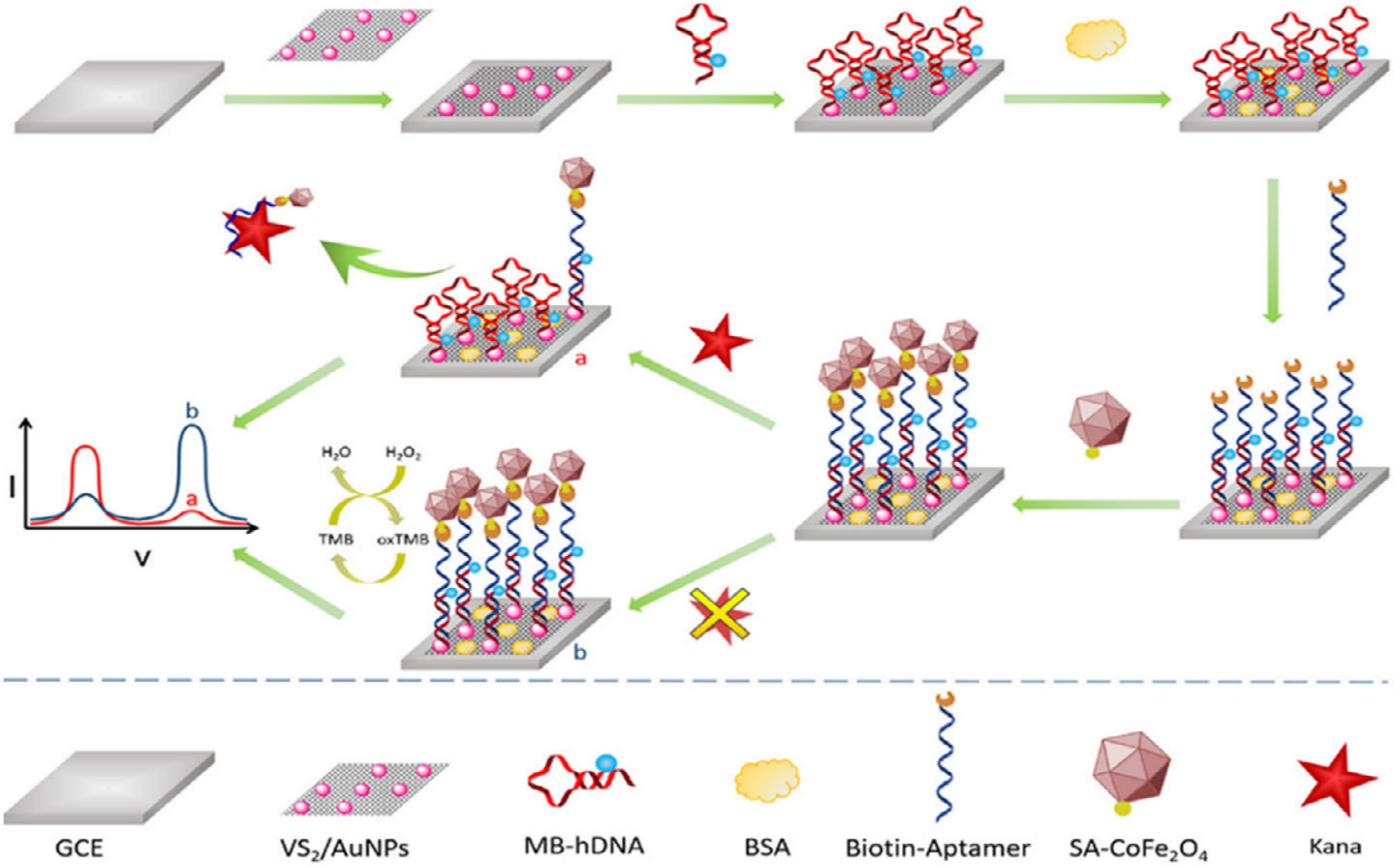
Illustrates preparation process and elctrochemical detetction strategy of detetction of kanamycin. Adapted with permission.^[^ ^70^ ^]^ Copyright 2020, American Chemical Society.

Enzyme mimics or artificial enzymes or nanozymes are synthetic or engineered materials designed to imitate the catalytic function of natural enzymes. They are often more stable than natural enzymes retaining their activity under harsh temperatures, pH, and chemical environment where proteins would denature. Peroxidase mimicking catalytic properties of CoFe_2_O_4_ and CeCu_2_O_4_ nanozymes^[^ ^70^, ^80^ ^]^ were utilized in an ultra‐sensitive electrochemical ratio metric biosensor as to determine the kanamycin in milk samples. Methylene blue labelled hairpin DNA and nanozyme CoFe_2_O_4_ were used in signal amplification, and to increase the sensitivity of the system, VS_2_/AuNPs nanocomposite electrodes were used (Figure 8).^[^ ^70^ ^]^

**Table 2:** List of the reported enzyme based electrochemical biosensors.

Biosensor type	Enzyme	Analyte	LOD ^a^ ^)^	Matrix	Label /Mediator	Response Time	Ref ^c^ ^)^
Voltametric	Penicillinase	Penicillin Tetracycline	10.5 µM 15.2 µM	Chicken Beef	–	–	^[^ ^72^ ^]^
	β‐lactamase	Benzylpenicillin	79 nM	Milk	–	–	^[^ ^73^ ^]^
	New Delhi metal‐β‐lactamase‐1	Penicillin G Ampicillin	0.174 ng/mL 0.35 ng/mL	Milk	–		^[^ ^74^ ^]^
	Penicillinase	Penicillin G	1.26 nM	–	–	10 sec	^[^ ^75^ ^]^
	Laccase	Tetracycline	89.4 µM	Honey Milk	–	–	^[^ ^76^ ^]^
	Class B β‐lactamase	Penicillin G	0.07 µM	Milk	Cobalt pthalocyanine	60 s	^[^ ^73^ ^]^
	Horseradish Peroxidase	Oxytetracycline	35 nM	Cow Serum	H_2_O_2_	–	^[^ ^77^ ^]^
	CoFe_2_O_4_ nanozyme	Kanamycin	0.5 pM	Milk	Methylene Blue	–	^[^ ^70^ ^]^
	CoCu_2_O_4_	Kanamycin	0.6 pM	Water	–	–	^[^ ^80^ ^]^
Amperometric	PBP3 conjugated with Glucose Oxidase	Piperacillin Cefuroxime Cefazolin	2.07 ng/mL 4.88 ng/mL 5.71 ng/mL	Serum	–	< 1 Hr	^[^ ^55^ ^]^
	β‐lactamase	Penicillin V	50 nM	PBS ^b^ ^)^ Milk	Multi‐ Walled Carbon Nanotubes	–	^[^ ^81^ ^]^
Impedimetric	DNA gyrase	Ciprofloxacin Norfloxacin	3.01 nM 30.1 nM	Water	–	–	^[^ ^82^ ^]^
	Penicillinase	β‐lactams	0.1 µM	Gastric Lavage	–	–	^[^ ^83^ ^]^

^a)^ Limit of Detection.

^b)^ Phosphate Buffer Saline.

^c)^ Reference.

#### 2.2.2 Amperometric Biosensors

**Figure 9:**
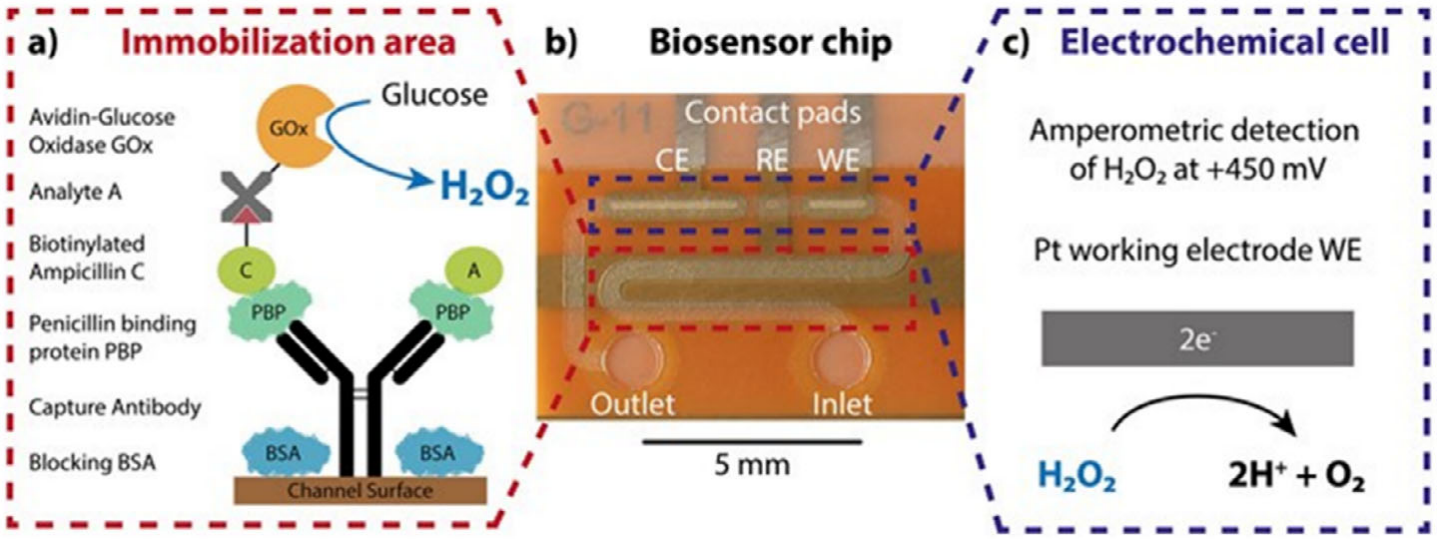
Illustration of electrochemical microfluidic platform: (a) Schematic of competitive β‐lactam assay based on PBP3 receptor and GOx enzyme. (b) Photograph of microfluidic integrated biosensing platform consisting of Counter electrode CE, the reference electrode RE and the working electrode WE.(c) Illustrates oxidation of hydrogen peroxide produced at Pt WE for amperometric detection inside the electrochemical cell. Adapted with permission.^[^ ^55^ ^]^ Copyright 2017, Nature.

Amperometric biosensors measure the amplitude of the reduction or oxidation current flow at a given potential for a fixed period.^[^ ^84^ ^]^ An amperometric biosensing platform based on competitive bioassay, PBP3 conjugated with Glucose Oxidase enzyme was used indirectly to quantify the presence of β‐lactams in human blood plasma (Figure 9).^[^ ^55^ ^]^ The system uses small volumes (1 µL) of reagent as well as sample. The effectiveness for personalised antibiotic therapy was demonstrated by monitoring pharmokinetics in patients undergoing surgery with β‐lactam antibiotics. Multiwalled Carbon Nanotubes (MWCNTs), hematein and β‐lactamase are sequentially deposited on glassy carbon electrode (GCE) by Layer by layer (LbL) assembly to develop a pH sensitive amperometric biosensor.^[^ ^81^ ^]^ Innovative biosensor designs display synergistic combination of bio‐recognition elements and nanomaterials for ultrasensitive antibiotic detection.

#### 2.2.3 Impedimetric Biosensors

Impedimetric sensing is a label‐free technique that is highly sensitive to interactions at the electrode‐electrolyte interface.^[^ ^85^, ^86^ ^]^ This pioneer work reports utilizing bacterial machinery‐DNA gyrase to detect quinolone class of antibiotics.^[^ ^82^ ^]^ Immobilization of DNA‐gyrase on carbon screen printed electrode modified with carboxylated carbon nanotubes demonstrates the benefits of the nanomaterial modification in electrochemical performance of electrode. Nanomaterials‐enhanced interfaces amplify electrochemical signals, providing ultrasensitive detection suited for critical applications. In this work Penicillinase is conjugated to chitosan‐modified platinum‐zinc oxide nanoparticles deposited on zinc hexacyanoferrate hybrid films on a fluorine‐doped glass electrode to achieve rapid, selective and sensitive detection of antibiotics in forensic and clinical samples.^[^ ^83^ ^]^

### 2.3 Thermometric Biosensors

The calorimetric changes resulting from enzymatic reactions were measured using thermometric biosensors such as thermistors^[^ ^87^ ^]^ by immobilising an enzyme in a microcolumn (Figure 11). In this work a nylon tubing is immobilized with penicillinase to allow continuous monitoring of benzylpenicillin in fermentation broth.^[^ ^88^ ^]^ A highly informative biosensor using the super resistant New Delhi metal‐1‐β‐lactamase (NDM‐1) as sensing element. The NDM‐1 biosensor platform is compatible with multiple β‐lactams and β‐lactamase inhibitors. It can differentiate clinical bacterial isolates expressing extended spectrum β‐lactamases (ESBLs), carbapenemases (CPs) *viz* metallo‐CPs and serine‐CPs in 23 clinical isolates with 100% accuracy. The biosensor can be used with antibiotic/inhibitor combinations, generating detailed activity profiles (Table 3).^[^ ^89^ ^]^

**Table 3:** List of the reported enzyme based thermometric biosensor.

Biosensor type	Enzyme used	Analyte	LOD^a^	Matrix	Label/Mediator	Response Time	Reference
Thermometric	Penicillinase	Penicillin‐G	^–^	Fermentation Broth	–	–	^[^ ^88^ ^]^
	New Delhi metal‐1‐β‐lactamase	Β‐lactams	–	Clinical Isolate	–	1 hr	^[^ ^89^ ^]^

## 3 Advances in Enzymatic Biosensors from 1960 to 2025

Enzyme‐ based biosensors have been transformed since their introduction in the mid‐twentieth century, from free enzymes integrated with selectively permeable membranes to integration of nanotechnology, advanced materials science, and advanced materials science, and synthetic biology (Figure 12).

### 3.1 First‐Generation Biosensors (1960s‐1970s): the Clarck Electrode and Early Enzymatic Detection

The enzyme‐based biosensor is pioneered by Leland C. Clark Jr's work in 1956 with introduction of Clark oxygen electrode for monitoring blood oxygen.^[^ ^90^ ^]^ First the enzymatic biosensors was reported in 1965^[^ ^91^ ^]^ with enzyme sandwiched in membrane between polarographic electrodes. The biosensor measured pH changes induced by enzymatic reaction by producing gluconic acid by glucose oxidation.

First‐generation biosensors were, however challenged in separating analyte and by products of enzyme activity^[^ ^92^ ^]^ limiting their clinical utility. To overcome this bottle neck various design principles, including enzyme immobilization techniques and the use of semipermeable membranes to enhance the selectivity.

### 3.2 Second‐Generation (1980s‐1990s): Redox Mediators for Enhanced Electron Transfer

Synthetic redox mediators like ferrocene derivatives were introduced in 1980s for efficient electron transfer between enzymes and electrodes. The mediated electron transfer from enzyme redox centre to the transducer, enabled operation at lower potentials and reducing interference from antioxidants like uric acid and ascorbic acid in biological samples. This advancement resulted in commercialisation of hand‐held glucose metres, revolutionizing diabetes management.

Shuttling of electrons from the enzyme to transducer led researchers to develop bi‐enzymatic systems for complex analytes.^[^ ^93^ ^]^ Redox mediators helped amplification the signal.^[^ ^54^, ^62^, ^66^, ^70^, ^74^, ^76^, ^80^ ^]^

### 3.3 Third‐Generation Biosensors (2000s‐Present): Nanomaterial for Direct Electron Transfer (DET)

Invention of Carbon nanotubes (CNTs)^[^ ^55^, ^81^, ^83^, ^94^ ^]^ and Graphene Oxide (GO) sheets^[^ ^95^, ^96^ ^]^ facilitated DET by providing high surface areas and conductive pathways, signal amplification and increasing sensitivity. Metalloenzymes with copper, zinc^[^ ^74^, ^89^ ^]^ or heme‐groups integrated with nanomaterials have proved effective for DET applications.

Further, advancement in microfabrication enabled multiplexed detection of various analytes. The first wearable enzymatic biosensor was introduced in 2010, including tattoo‐based glucose monitors utilizing glucose oxidase‐functionalized prussian blue nanoparticles.^[^ ^97^ ^]^ Enzymes being biomolecule are susceptible to change in pH and temperature induced denaturation and hence lose their stability to be functional over time. An advancement to this bottleneck is introduction of nanomaterials with catalytic activity and efficiency as like natural enzymes, called Nanozymes (Figure 13).

## 4 Surveillance Studies on Antibiotic Residues

Biosensors have remarkably advanced the analytical techniques used for detection of antibiotic residues. The effectiveness of biosensors pivots on their sensitivity, specificity and reproducibility. However, for on‐site application of biosensors is challenged due to various reasons like matrix‐related variability and environmental interference. Meng et al.^[^ ^89^ ^]^ a patient centric enzyme‐based thermometric biosensor to detect β‐lactamase AMR status in an hour with minimal sample preparation eliminating culturing and sample pretreatment. The NDM‐1 biosensor had 16 folds better sensitivity than commercially available ESBL Nordmann/Dortet/Poirel (NDP) and 8 times than Carba Nordmann/Poirel (NP) assay (Figure 14).

**Figure 10:**
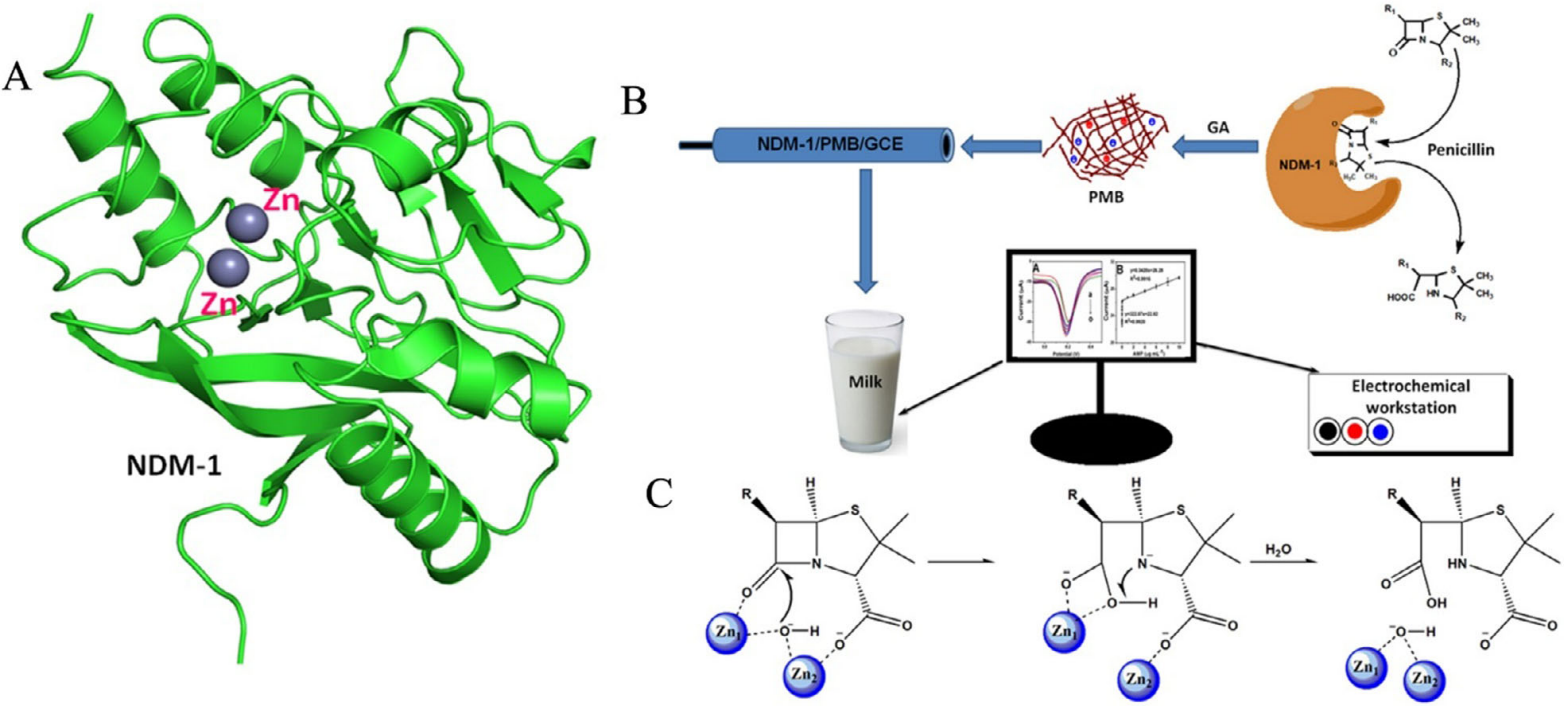
Illustrates NDM‐1 β‐lactamase structure (A) and schematics of the preparation of electrochemical immunosensor based on NDM‐1 β‐lactamase(B). Schematics of penicillin reaction at the active site of di‐Zinc metallo β‐lactamase NDM‐1 (C). Adapted with permission.^[^ ^74^ ^]^ Copyright 2020, Yi Xiu, Ruiping Luo, Baoqing Han, Lu Liu and Hongsu Wang.

**Figure 11:**
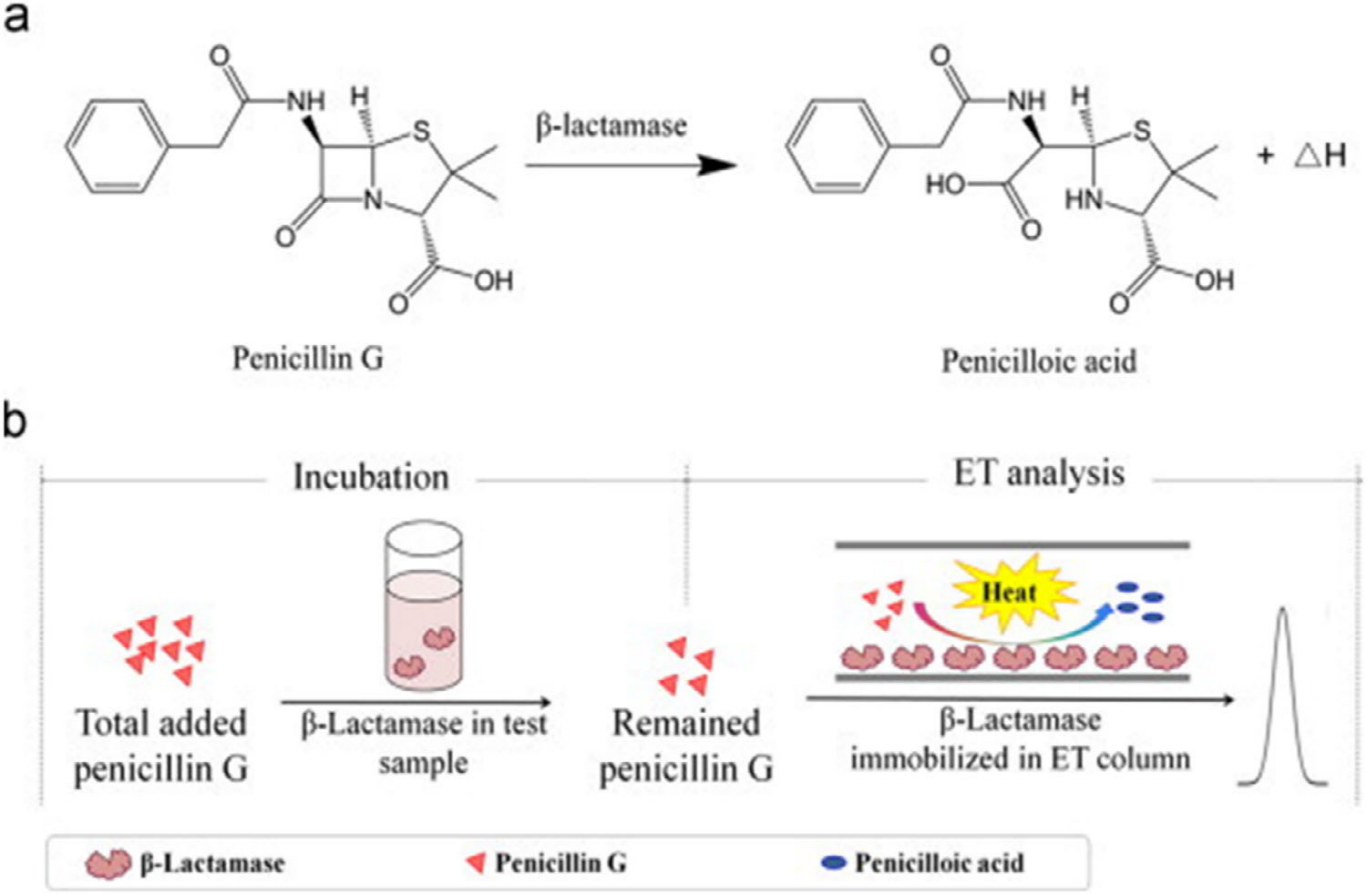
Schematics of (a) hydrolysis of β‐lactam ring in Penicillin G by β‐lactamase and (b) principle of thermometric analysis procedure. Adapted with permission.^[^ ^56^ ^]^ Copyright 2013, Elsevier.

**Figure 12:**
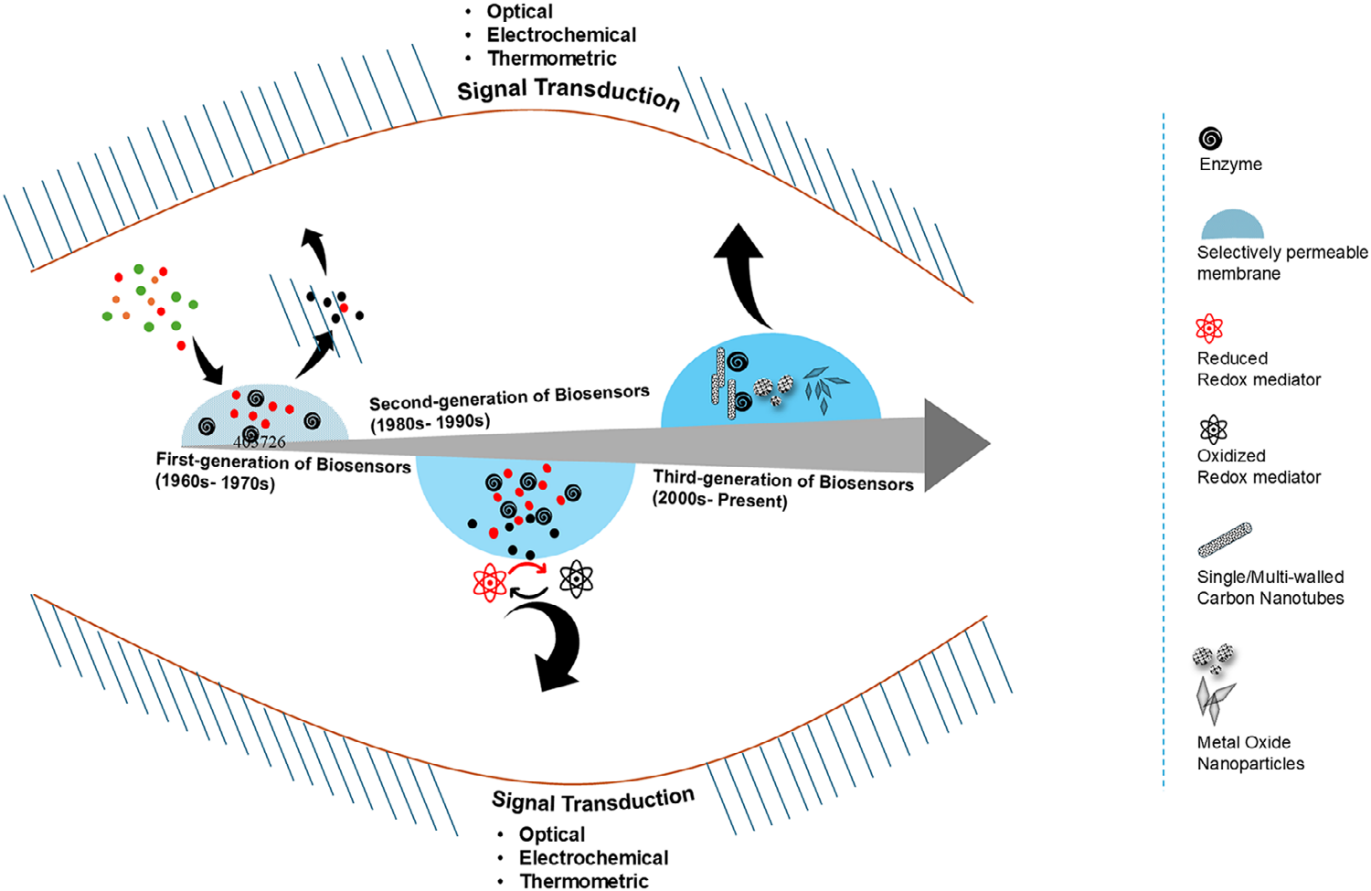
Illustration of classification of enzyme based biosensors from first to third generation.

**Figure 13:**
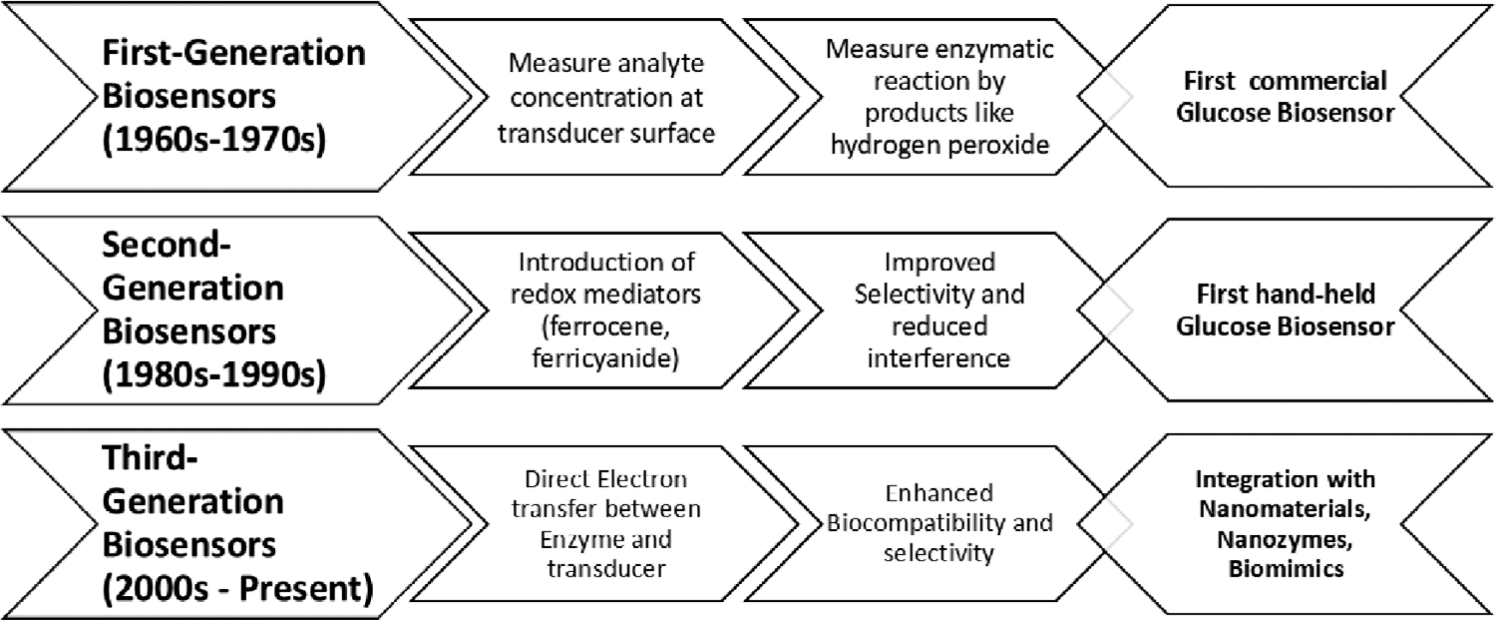
Categorization of enzymatic biosensors, as generations, based on mechanism of electron transfer from enzyme to transducer for optical, electrochemical and thermometric signal.

**Figure 14:**
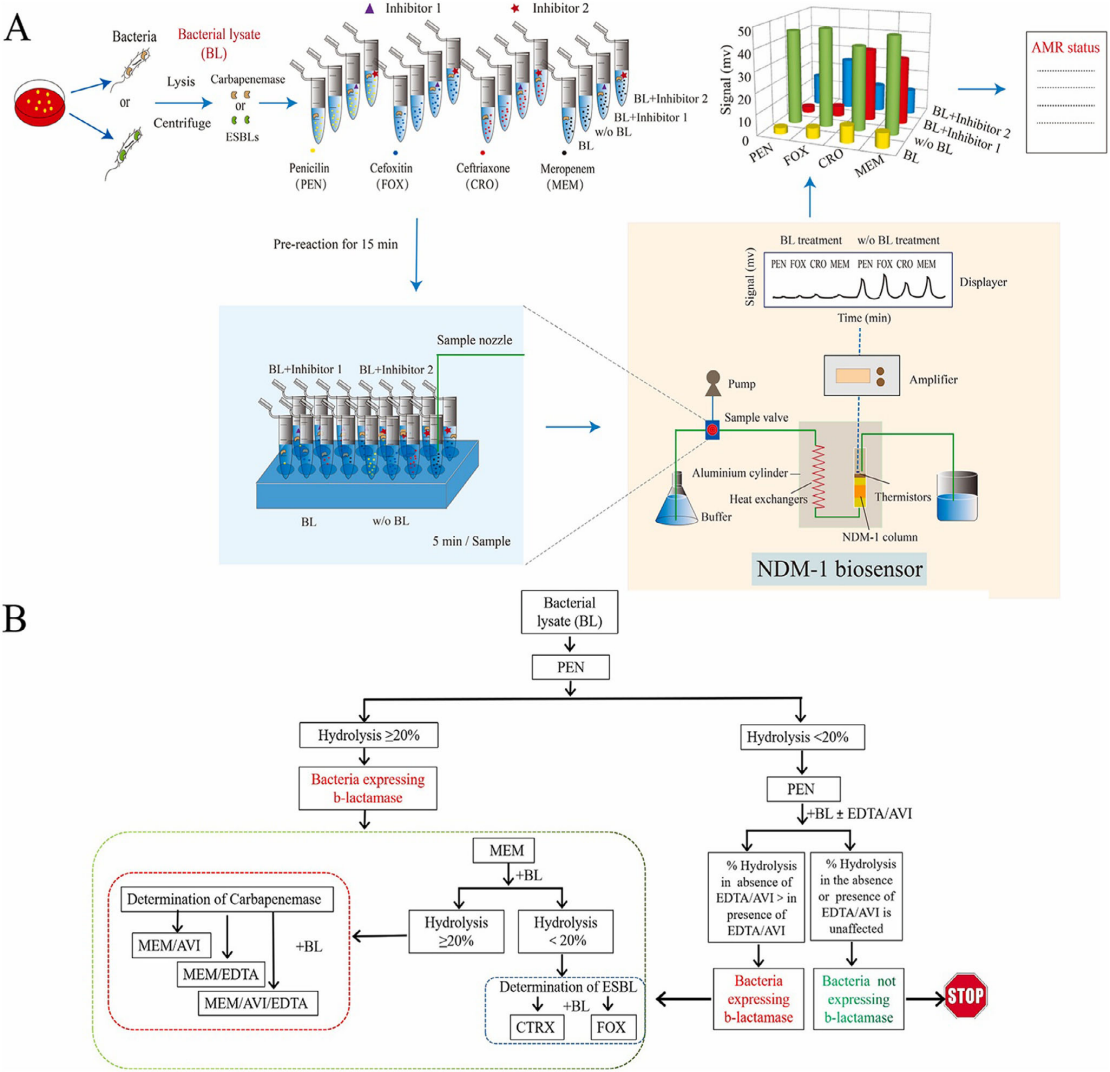
Schematics of the novel AMR assay strategy and an algorithm to identify bacteria expressing ESBL and CP. A: Clinical bacterial samples are lysed, and individual aliquots are mixed in parallel with a panel of antibiotics in the presence or absence of inhibitors, followed by thermometric NDM‐1 biosensor analysis. The heat signal generated from hydrolysis of the injected antibiotic by the immobilized NDM‐1 enzyme is transformed into an electronic signal by the thermistor. This represents the amount of antibiotic that remains in the sample which is inversely proportional to the β‐lactamase activity present in the sample. These signals are combined to generate a profile which is compared with that of known β‐lactamases to determine AMR status, e.g. the presence of ESBL and/or CP. B: Based on the detection principle shown in Figure 1A, a practical algorithm was designed to simultaneously identify bacteria expressing ESBL and CP within 1 h. Adapted with permission.^[^ ^89^ ^]^ Copyright 2021, Elsevier.

## 5 Summary and Outlook

Enzymatic biosensors, despite their remarkable success in various biomedical and environmental applications, remain underutilized for antibiotic detection. This under exploration stems primarily from the intrinsic challenges associated with enzyme stability and sensitivity. Many enzymes exhibit high sensitivity toward changes in temperature, pH, and the presence of interfering substances, factors commonly encountered in real world samples such as clinical fluids (blood, sputum, urine), food and environmental water. In these complex matrices, an array of molecules can directly inhibit or alter enzyme activity, leading to unreliable results. Enzymes, by their biological nature, can have varying affinity and selectivity for antibiotic molecules. This variability can result in cross‐reactivity or false positives, especially when unknown or structurally similar compounds are present. These challenges are further compounded by extremely low concentrations at which antibiotics may be present in clinical or environmental samples, thus demanding ultrasensitive detection capabilities

Furthermore, lack of standardized protocols for sample preparation, enzymatic assay procedures, and data interpretation means that results obtained in different laboratories can vary significantly, hampering reproducibility and slowing regulatory acceptance. In environmental monitoring, the situation is complicated by interference from natural organic matter, heavy metals, and other pollutants found in water, which can further diminish the selectivity and sensitivity of enzymatic biosensors.

Despite these obstacles, the potential benefits of enzymatic biosensors for antibiotic detection are enormous. They offer rapid, real‐time, and cost‐effective analysis which are crucial for timely clinical decisions and for addressing the growing crisis of antibiotic resistance and environmental contamination.

To overcome the operational and functional barriers of enzyme‐based biosensors, cutting‐edge solutions must be integrated.

- Nanotechnology and protein engineering allows for the stabilization and functionalization of enzymes, enhancing both their robustness and catalytic efficiency, even under challenging conditions.
- Microfluidic platforms can provide precise sample handling and enable high‐throughput, multiplexed analysis, minimising sample volume and reducing the risk of contamination.
- Smart signal amplification and transduction strategies can increase the detection sensitivity and reliably identify antibiotics at trace levels.

In conclusion, while challenges remain, the future of enzymatic biosensors for antibiotic detection is highly promising with researchers harnessing the integration of emerging interdisciplinary technologies. By overcoming the bottlenecks of stability, specificity, sensitivity, and standardization, it will be possible to create robust, field deployable biosensors that provide continuous, real‐time monitoring of antibiotics in clinical, food, and environmental samples. Such advancements are essential for safeguarding public health, supporting antibiotic stewardship and ensuring environmental safety in a world increasingly threatened by antimicrobial resistance.

## Conflict of Interests

The authors declare no conflict of interest.

## Data Availability

Data sharing is not applicable to this article as no new data were created or analyzed in this study.
